# Functional Heterogeneity of Bone Marrow Mesenchymal Stem Cell Subpopulations in Physiology and Pathology

**DOI:** 10.3390/ijms231911928

**Published:** 2022-10-07

**Authors:** Kaiting Ning, Baoqiang Yang, Meng Chen, Guigui Man, Shuaiting Liu, Dong-en Wang, Huiyun Xu

**Affiliations:** 1 Key Laboratory for Space Bioscience and Biotechnology, School of Life Sciences, Northwestern Polytechnical University, Xi’an 710072, China; 2 Research Center of Special Environmental Biomechanics and Medical Engineering, Northwestern Polytechnical University, 127 West Youyi Road, Xi’an 710072, China

**Keywords:** functional heterogeneity of BMSCs, physiological status, pathological-related BMSC subpopulation

## Abstract

Bone marrow mesenchymal stem cells (BMSCs) are multi-potent cell populations and are capable of maintaining bone and body homeostasis. The stemness and potential therapeutic effect of BMSCs have been explored extensively in recent years. However, diverse cell surface antigens and complex gene expression of BMSCs have indicated that BMSCs represent heterogeneous populations, and the natural characteristics of BMSCs make it difficult to identify the specific subpopulations in pathological processes which are often obscured by bulk analysis of the total BMSCs. Meanwhile, the therapeutic effect of total BMSCs is often less effective partly due to their heterogeneity. Therefore, understanding the functional heterogeneity of the BMSC subpopulations under different physiological and pathological conditions could have major ramifications for global health. Here, we summarize the recent progress of functional heterogeneity of BMSC subpopulations in physiology and pathology. Targeting tissue-resident single BMSC subpopulation offers a potentially innovative therapeutic strategy and improves BMSC effectiveness in clinical application.

## 1. Introduction

BMSCs are implicated in various basic functions, such as proliferation, differentiating into multiple bone marrow cell types, supporting hematopoiesis, and regulating immunity throughout our whole life [1]. Previous findings have reported that BMSCs are heterogeneous mixtures of diverse progenitor cell populations [2,3,4,5]. The invention of flow cytometry [6] enables researchers to be able to isolate and identify BMSC subpopulations with certain cell surface proteins and genetic fluorescence-tagged cell types, according to self-renewal ability and osteo-adipogenic potential [7,8,9,10]. In the late 2000s, the Matsuzaki group first isolated the platelet-derived growth factor receptor (PDGFR) α positive subpopulation and PDGFRβ positive subpopulation from adult mouse bone marrow via flow cytometry [11,12]. Over time, researchers developed lineage tracing technology to trace more BMSC subpopulations labels in vivo, including paired related homeobox 1 (Prx1), Nestin, and myxovirus resistance 1(Mx-1), and leptin receptor (LepR) et al. [13,14]. Later, advances in single-cell RNA sequencing (scRNA-seq) provided further insight into the heterogeneity of BMSC subpopulations in healthy adults and challenged mice [15,16,17,18,19], and scRNA-seq data can show us the division of hierarchical relationships between different BMSC subpopulations [17]. The development of these technologies added new dimensions to the heterogeneous study of BMSC subpopulations.

**Figure 1:**
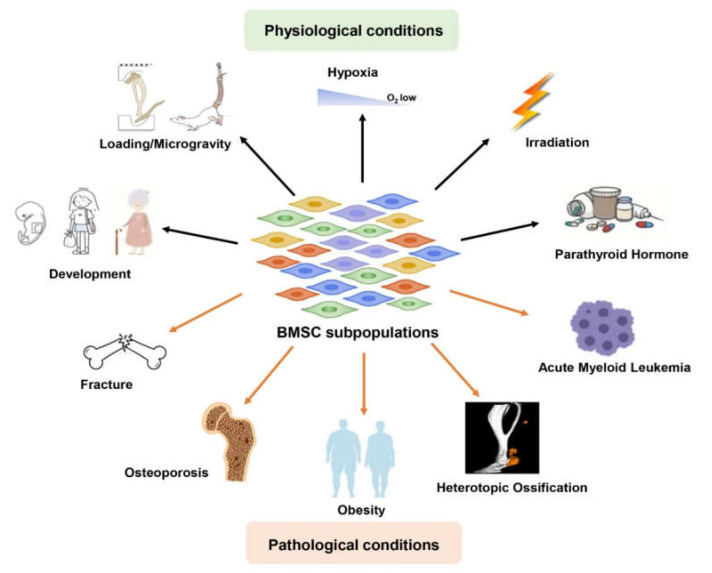
Functional heterogeneity of BMSC subpopulations in physiology and pathology. Physiological conditions include development (embryonic development, adulthood, and aging) and various environmental stresses like loading, microgravity, hypoxia, irradiation, and parathyroid hormone (PTH). Pathological conditions include fracture, osteoporosis, obesity, heterotopic ossification, and acute myeloid leukemia (AML).

More recent evidence indicated that heterogeneous BMSC subpopulations exhibited distinctive differentiation potentials and functions [20,21]. Meanwhile, functional heterogeneity of specific BMSC subpopulations contributes to pathological processes, and significant changes in specific subpopulations are often covered up by bulk analysis of the total BMSCs. Additionally, BMSCs possess therapeutic potential for the repair and regeneration of damaged tissues; but targeting total BMSCs is not always effective. Therefore, understanding the heterogeneity of the BMSC subpopulations under different physiological and pathological conditions could make a major contribution to health. In this review, we summarize the functional heterogeneity of BMSC subpopulations in the different development stages (e.g., embryonic development, adulthood, and aging), various environmental stresses (like loading, microgravity, hypoxia, irradiation as well as parathyroid hormone (PTH)) (Figure 1 and Table 1), and different pathological conditions (such as fracture, osteoporosis, heterotopic ossification, obesity as well as acute myeloid leukemia (AML)) (Figure 1). Targeting the tissue BMSC subpopulations offers a potentially innovative therapeutic strategy and improves BMSC effectiveness in clinical application.

## 2. Functional Heterogeneity of BMSC Subpopulations in Physiology (Table 1)

### 2.1. Development

#### 2.1.1. Embryonic Development

Diverse BMSC subpopulations are involved in the embryonic period [22,23,24,25,26,27,28]. CD105 positive BMSC subpopulations can be detected as early as embryo day 13 (E13) [22], which supports hematopoietic generation by endochondral ossification in fetal mice [22,29]. Simultaneously, another finding has reported that E13.5 Grem1-Cre traces almost the entire embryonic mesenchyme and primary spongiosis, which overlaps with metaphyseal anatomical localization of adulthood Grem1-expressing cells, suggesting the subpopulation has chondrogenic potential [14]. Moreover, 6C3-supportive stromal cells and LepR^+^ cells in the long bone of E17.5 mice co-emerge with HSCs, underscoring the supporting hematopoietic niche of these two subpopulations [5,22]. In addition to different time points of embryonic development, BMSCs from differential bone regions also exhibited heterogeneity. For example, Maruyama et al. proposed that axis inhibition protein 2 positive (Axin2^+^) subpopulations are restricted to the midline of craniofacial sutures, mainly traced cranial skeletal stem cells (SSCs) throughout all stages of development in mice crania, but which are virtually absent in long bones [30,31,32], while Glioma-associated oncogene 1(Gli1)-expressing cells share characteristics with long bone BMSCs and are abundantly present along with the whole craniofacial sutures, but both subpopulations are thought to promote growth and regeneration [16,32]. The Sox9-expressing BMSC subpopulation is involved in initial cartilage templates formation, and tracing the subpopulation from early embryonic stages found that it marked osteochondrogenic, adipogenic, and stromal cells like CAR (CXCL12-abundant reticular) cells of adult bone marrow [33]. Similarly, Osterix (Osx)^+^ cells reside in bone marrow stroma during fetal development, and further form perivascular, osteogenic, and adipogenic cells [25,34]. Prx1 is highly expressed by limb bud mesenchymal progenitors during long bone development [35], and genetic lineage tracing by Prx1-Cre labels all skeletal lineage cells in long bones, including skeletal stem/progenitor cells (SSPCs), osteoblasts, osteocytes, chondrocytes, and adipocytes [36].

Early studies of BMSC subpopulations on embryonic bone development mainly focused on mice. Most recently, Yue and his colleagues explored spatiotemporal ontogeny of human embryonic limb and long bones during early skeletogenesis through scRNA-seq [37]. They revealed distinguished heterogeneity of mesenchyme cells within different human limb bud regions, like mesenchyme and epithelium, as well as aligning them along the proximal–distal and anterior–posterior axes [37]. Moreover, they proposed that osteo-chondrogenic populations first appeared in the core limb bud mesenchyme, which derived multiple populations of stem/progenitor cells in embryonic long bones through endochondral ossification [37]. Importantly, a perichondrial embryonic skeletal stem/progenitor cell (eSSPC) population marked by adhesion molecule 1 (CADM1^+^) and podoplanin (PDPN^+^) was identified, which could self-renew and give rise to the osteochondral lineage cells, but not adipocytes or hematopoietic stroma. Interestingly, neural crest-derived cells with similar phenotypic markers with limb buds-derived eSSPC were also found in the sagittal suture of human embryonic calvaria [37].

In sum, various mouse BMSC subpopulations reside in different bone regions and exhibit multipotential differentiation and support hematopoiesis during the embryonic period (Table 1). In detail, the BMSC subpopulations labeled CD105^+^, 6C3^+^, and LepR^+^ cells types support mice hematopoiesis; Axin2^+^ and Gli1^+^ both can label cranial SSCs in mice, contributing to growth and regeneration; Germ1^+^ and Sox9-expressing BMSC populations locate in cartilage templates, while Osx^+^ cells are located in bone marrow, and both further differentiate into osteochondrogenic, adipogenic, or stromal cells. Prx1^+^ cells are highly expressed by limb bud mesenchymal progenitors and following this, differentiate into all kinds of long bone cells. Additionally, CADM1^+^ PDPN^+^ eSSPC contributes self-renew and generates the osteochondral lineage cells, but not adipocytes or hematopoietic stroma during human embryonic development.

**Table 1:** Functional heterogeneity of BMSC subpopulations in Physiology.

Physiological Conditions	BMSC Subpopulations	Functions	References
**Development**			
Embryonic development	Mice: CD105^+^, Grem1^+^, 6C3^+^, LepR^+^, Axin2^+^, Gli1^+^, Osx^+^, Sox9^+^, Prx1^+^ Human: CADM1^+^ PDPN^+^	Supports hematopoiesis: CD105^+^, 6C3^+^, and LepR^+^	[5,22,29]
		Promotes growth and regeneration: Axin2^+^ and Gli1^+^ (label cranial SSCs)	[16,30,31,32]
		Multi-lineage differentiation: Osx^+^ (bone marrow), Grem1^+^ and Sox9^+^ (cartilage templates), Prx1^+^ (limb bud)	[25,33,34,35,36]
		Promotes self-renewal and generates the osteochongenesis but no adipocytes and no hematopoietic supportive function: CADM1^+^ PDPN^+^	[37]
Adulthood	Mice: PDGFRα^+^ (PDGFRa^+^ Sca-1^−^ CD45^−^ Ter119^−^), Nestin^+^ (Nestin-GFP*^low^* cells), Osx^+^, LepR^+^, Acan^+^, Mx1^+^, Prx1^+^, PTHrP^+^, Grem1^+^, Gli1^+^, mpMSCs	Promote osteogenesis and support the hematopoiesis: PDGFRα^+^, Nestin^+^, Osx^+^, LepR^+^, Acan^+^ Mx1^+^, Sca1^+^, CD105^+^	[11,38,39,40,41,42,43,44,45]
		Label specific bone regions: bone marrow of limb bones (Prx1^+^); chondrocytes of the resting zone in the growth plate of long bones (PTHrP^+^); metaphyseal areas (Grem1^+^, Gli1^+^, mpMSCs)	[46,47,48,49,50,51,52]
	Human: CD105^+^, CD140a^+^, CD73^+^, CD90^+^, STRO1^+^, CD271^+^, CD44^+^, CD146^+^ CD271^+^PDGFRα*^low^*, STRO-1^+^, CD45^−^Ter119^−^ Tie2^−^ Thy1^−^ 6C3^−^ CD51^+^, PDPN^+^ CD73^+^ CD164^+^ CD235^−^ CD45^−^ CD146^−^ Tie2^−^ CD31^−^, FGFR2^+^, FGF5^+^, PLAT^+^ VCAM1^+^	Exhibits high CFU-F ability and multi-lineage differentiation potential: CD105^+^, CD140a^+^, CD73^+^, CD90^+^, STRO-1^+^, CD271^+^, CD44^+^, PDPN^+^ CD73^+^ CD164^+^ CD235^−^ CD45^−^ CD146^−^ Tie2^−^ CD31^−^, FGFR2^+^, FGF5^+^, PLAT^+^ VCAM1^+^	[7,8,9,10,15,22,29,53,54,55,56,57,58]
		Supports hematopoiesis: CD146^+^, CD146^+^ CD271^+^ PDGFRα*^low^*, STRO-1^+^	[29,53,54,55]
		Label specific bone regions: bone cartilage stromal (CD45^−^ Ter119^−^ Tie2^−^ Thy1^−^ 6C3^−^ CD51^+^); hypertrophic zones of the growth plate (PDPN^+^ CD73^+^ CD164^+^ CD235^−^ CD45^−^ CD146^−^ Tie2^−^ CD31^−^)	[15,22,56,57]
		Specific functional subpopulation of UC-MSCs: high immune response/regulatory activities (group 1 of UC-MSCs); bone and cartilage growth related group 2 of UC-MSCs	[59]
Aging	Mice: Sca1^+^, Prx1^+^, LepR^+^, LepR^+^ Notch3^+^, LepR^+^ MALPs	Decreases number and impairs paracrine support for hematopoiesis: Sca1^+^, Prx1^+^, LepR^+^, LepR^+^ Notch3^+^	[60,61,62,63]
		Increases the number and promotes adipogenesis: LepR^+^ MALPs	[19]
	Human:CD29^+^ CD44^+^ CD90^+^ CD105^+^ CD34^−^ CD45^−^ HLA-DR^−^	Self-renewal related subpopulation: high expression, *CDCA5*, *MYBL2*, *FAM64A*, *CENP-A*, *PAQR4*, *Asf1b*, *CAF-1*, *HMGB2*	[64,65,66,67,68,69,70,71,72]
		Multidirectional differentiation-related subpopulation: high express *TGM2*, *COL11A1*, *NEAT1*, *Type V collagen*	[64,73,74,75,76]
		Immune regulation and damage repair related subpopulation: high express *Cyba*, *TIMP-1*, *ANXA1*, *LUM*, *DPT*, *ERp44*, and *HSPA5*	[64,77,78,79,80,81,82,83]
**Environmental Stresses**			
Loading	Sca-1^+^ Prx1^+^, Osx^+^, CXCL12^+^, LepR^+^ osteolectin^+^	Responds to loading and participating in bone formation	[11,38,84,85,86,87,88,89]
Microgravity	Sca^+^ CD90.2^+^, Lin^−^ LepR^+^, LepR^+^ osteolectin^+^	Declines number and exhibits more quiescence and lower bone anabolism	[89,90,91]
Hypoxia	CD13^+^ CD29^+^ CD44^+^ CD73^+^ CD90^+^ CD105^+^ CD151^+^ CD34^−^, PDGFRα^+^, LepR^+^, SP7^+^, 7AAD^−^ CD45^−^ Ter119^−^ Tie2^−^ CD51^+^ CD105^−^ CD90.2^−^ CD249^−^ CD200^−^	Exhibits high proliferative activity: CD13^+^ CD29^+^ CD44^+^ CD73^+^ CD90^+^ CD105^+^ CD151^+^ CD34^-^, PDGFRα^+^ and LepR^+^	[92,93]
		Osteogenic and chondrogenic differentiation: SP7^+^ (also know as Osx^+^), 7AAD^−^ CD45^−^ Ter119^−^ Tie2^−^ CD51^+^ CD105^−^ CD90.2^−^ CD249^−^ CD200^+^	[94,95]
Irradiation	Mice: LepR^+^, Nestin^+^, CD73^+^ NGFR*^high^*, LepR^+^ MALPs	Declines number after irradiation: LepR ^+^, Nestin ^+^	[96]
		Expansion, supports hematopoietic and bone marrow repair: CD73^+^ NGFR*^high^*, LepR^+^ MALPs, LepR^+^ BMSCs with high expression of Npdc1/ Hoxb2	[19,63,96]
	Human: CD73^+^ CD90^+^ CD105^+^ CD14^−^ CD34^−^ CD45^−^ HLA-DR^−^	Exhibits senescence and impairs immunomodulation capacity: CD73^+^ CD90^+^ CD105^+^ CD14^−^ CD34^−^ CD45^−^ HLA-DR^−^	[97]
PTH	LepR^+^, LepR^+^ Runx2-GFP*^low^*	Promotes osteogenic differentiation by promoting osteolectin expression or increasing numbers of type H endothelial cells	[98,99,100,101,102]

#### 2.1.2. Adulthood

From postnatal to adulthood, there are still various bone-resident BMSC subpopulations to maintain bone homeostasis. While the essential role of BMSCs in the postnatal development of the skeleton has been generally established, identifying pure BMSC subpopulations remains very important. Here, we mainly discuss the functional heterogeneity of BMSC subpopulations in adult bone development both in mice and humans.

##### Mouse BMSCs

Mouse BMSCs have been prospectively identified in the perivascular [53,103], while the populations usually lack expression of hematopoietic and endothelial markers but have positive expression of PDGFRα [11,38,39]. Later, Morikawa and Omatsu identified two distinctive subpopulations of PDGFRα^+^ BMSCs, PDGFRα^+^ Sca-1^+^ CD45^−^ Ter119^−^ BMSCs and PDGFRα^+^ Sca-1^−^ CD45^−^ Ter119^−^ BMSCs [11,38]. The former one resides primarily around arterioles but does not express the HSC niche factor Cxcl12, while the latter one resides primarily around sinusoids and expresses high levels of Cxcl12 to support hematopoiesis [11,38]. Similarly, another study found that Nestin-GFP^+^ BMSCs are heterogeneous, including both Nestin-GFP*^high^* cells that localize mainly around arterioles and Nestin-GFP*^low^* cells that localize mainly around sinusoids; both subpopulations can osteogenesis but Nestin-GFP*^low^* cells secret more Cxcl12 [40,41]. Additionally, a previous study of Yue showed that LepR also marks SSPCs which localize in the perivascular region of the adult bone marrow [5] and promote osteogenesis [42], supporting the hematopoietic microenvironment by secreting high levels of Cxcl12 [5,43,44,45]. It has to be noticed that LepR^+^ BMSCs overlap with Nestin-GFP*^low^* cells [40,41], Cxcl12-abundant reticular (CAR) cells [44], and Osterix^+^ BMSCs [39]. In contrast to LepR^+^ cells, Mx-1-Cre cells overlap with Nestin-GFP, PDGFRα, Sca1, and CD105 BMSCs and give rise to most of the osteoblasts formed in adult bone marrow, but Mx-1 also robustly labels hematopoietic cells, meaning that this marker could not be specific to selection of osteogenic progenitors population [39]. Moreover, Prx1 also is another important BMSC marker and overlaps with LepR^+^ stromal cells as well as LepR^−^ osteoblasts and chondrocytes within the bone marrow of limb bones, but not in the axial skeleton [46]. Most recently, Shu et al. found that Acan^+^ BMSCs and LepR^+^ BMSCs controlled bone formation before and after adolescence, respectively, and Acan^+^ BMSCs mediate bone lengthening, while LepR^+^ BMSCs regulate bone thickening [47]. In addition to the heterogeneity of BMSCs at different bone development stages, the heterogeneity of BMSCs from different bone regions has also received increasing attention. A recent publication proposes parathyroid hormone-related protein (PTHrP) as a label for chondrocytes of the resting zone in the growth plate of long bones which descend from a PTHrP^+^ SSCs [48]. Other findings also revealed that both Grem1^+^ cells and Gli1^+^ cells mainly reside in metaphyseal areas, Grem^+^ cells are non-adipogenic, while Gli1^+^ cells contribute to osteochondrogenesis and adipogenesis in vivo [49,50]. Later evidence suggested that Gli1 is also seen in many mature bone cell types (like, Osx, Col1) not uniquely marking an homogeneous BMSC population [50]. Recently, Sivaraj et al. characterized the heterogeneity of BMSCs during skeletal development. They identified that BMSCs from metaphysis and diaphysis have distinct properties, and the subpopulation of metaphyseal MSCs (mpMSCs) has multi-lineage differentiation potential to give rise to bone cells and LepR^+^ BMSCs, and transcription factors of platelet-derived growth factor B (PDGF-B) and Jun-B control BMSC osteogenesis [51]. Aside from growth plate and metaphyseal areas, many MSCs also reside along the periosteum, where they can quickly get “activated” upon injury and facilitate proper fracture healing [52].

Taken together, different mouse BMSC subpopulations exhibit distinctive locations and more complicated functional heterogeneity for osteogenesis and hematopoiesis-supporting in the adult murine bone marrow (Table 1). Perivascular PDGFRα^+^ (PDGFRα^+^ Sca-1^−^ CD45^−^Ter119^−^), Nestin^+^ (Nestin-GFP*^low^* cells), Osx^+^, LepR^+^, Acan^+^ are all labeled postnatal mouse BMSCs which all can promote osteogenesis and secret high-level Cxcl12 to support the hematopoietic microenvironment. Mx-1-Cre cells reside in the adult bone marrow which overlaps with Nestin-GFP, PDGFRα, Sca1, CD105 BMSCs and gave rise to most of the osteoblasts; Mx-1 also robustly labels hematopoietic cells; additionally, Prx1^+^ labels for BMSCs of limb bones; PTHrP^+^ labels for chondrocytes of the resting zone in the growth plate of long bones; Grem1^+^ cells mainly reside in metaphyseal areas; Gli1 labels early postnatal multipotent progenitor cells in the metaphyseal region of long bones but does not uniquely mark a homogeneous BMSC population, all involved in osteogenesis of specific bone regions. Metaphyseal MSCs (mpMSCs) have multi-lineage differentiation potential and fate controlled by transcription factors PDGF-B and Jun-B.

##### Human BMSCs

Taking advantage of flow cytometry and scRNA-seq application, research on the heterogeneity of human BMSC subpopulations has also made good progress. In earlier studies, CD45 negative non-hematopoietic fibroblast colony-forming cells have been confirmed, and copious markers, including CD105, CD140a, CD73, CD90, STRO-1, CD271, and CD44 [7,8,9,10]. Another study reported that the CD146^+^ MSC subpopulation resides around sinusoidal blood vessels in the ossicles in the human bone marrow which can differentiate into osteogenic, chondrogenic, and adipogenic cells in culture and give rise to the bone upon transplantation in vivo, express HSC niche factors, and form bony ossicles that become invested with hematopoietic bone marrow [29,53]. Subsequent studies identified CD271^+^ PDGFRα*^low^* and the stromal cell antigen 1(STRO-1^+^) turned out to most efficiently select for perivascular residing SSC-like cells that are also able to maintain human HSCs for an extended time in culture [54,55]. Groundbreaking experiments by Chan et al. have transplanted human single cells, termed bone cartilage stromal progenitor (BCSPs; CD45^−^ Ter119^−^ Tie2^−^ Thy1^−^ 6C3^−^ CD51^+^), to a renal capsule of mice, and confirmed the CD45^−^ Ter119^−^ Tie2^−^ Thy1^−^ 6C3^−^ CD51^+^ BCSPs were bone fide stem cells in vivo [22]. Currently, Chan et al. identified a highly purified human skeletal stem cell (hSSC) distinct from the reported CD146-positive SSCs; and found in fetal and all adult stages throughout different skeletal sites but specifically enriched in the hypertrophic zones of the growth plate labeled by PDPN, CD73, CD164 and lacking expression of CD235, CD45, CD146, Tie2, and CD31 [56], and the hSSC populations can give rise to osteoprogenitor cell types, never producing fat [15,57]. Moreover, Liu et al. have analyzed the BMSC heterogeneity of one healthy subject hip through sc-RNAseq, and they divided the BMSCs into three subpopulations [58]. Subpopulation A was characterized by the high expression of fibroblast growth factor receptor 2 (FGFR2) which was involved in osteogenesis; Subpopulation B expressed higher levels of fibroblast growth factor 5 (FGF5) which could increase osteogenic differentiation of MSCs; and Subpopulation C was characterized by high expression of plasminogen activator tissue type (PLAT) and vascular adhesion molecule 1(VCAM1) which promoted angiogenesis [58]. Similarly, Zhang et al. have proposed umbilical cord mesenchymal stem cells (UC-MSCs) having two subpopulations via sc-RNAseq [59]. Group 1 MSCs are enriched in the expression of genes in immune response/regulatory activities (e.g., *TNFα*, *IL17*, *TLR*, *TGFβ*, infection, *NOD*, *NF-κB*, and *PGE* pathways), muscle cell proliferation and differentiation, stemness, and oxidative stress while group 2 MSCs are enriched with gene expression in extracellular matrix production, bone, and cartilage growth as well glucose metabolism [59].

In sum, human BMSC subpopulations possess more complex cell surface markers, exhibit specific bone regions distributions and different differentiation potential, and the subpopulations from the hip and umbilical cord show distinctive functional heterogeneity (Table 1). In detail, human BMSCs express copious markers, including CD105, CD140a, CD73, CD90, STRO-1, CD271, and CD44 et al., which exhibited variable CFU-F ability and multi-lineage differentiation potential. CD146^+^, CD271^+^ PDGFRα*^low^*, and STRO-1^+^ BMSC subpopulations contribute to HSC niche stability. CD45^−^ Ter119^−^ Tie2^−^ Thy1^−^ 6C3^−^ CD51^+^ labels BCSPs; while PDPN^+^ CD73^+^ CD164^+^ CD235^−^ CD45^−^ CD146^−^ Tie2^−^ CD31^−^ labels BMSCs, residing in hypertrophic zones of the growth plate, and these subpopulations just give rise to osteoprogenitor cell types, never producing fat. In addition, BMSCs of the healthy subject can be divided into FGFR2^+^; FGF5^+^ BMSCs and PLAT^+^ VCAM1^+^ BMSCs promote osteogenesis and angiogenesis, respectively. Similarly, UC-MSCs also can be divided into immune response/regulatory activities related to MSC group 1 and bone and cartilage growth-related MSC group 2. Importantly, human BMSC subpopulations showed distinctive markers with mouse BMSCs.

#### 2.1.3. Aging

Both the number and function of BMSCs decline dramatically with aging. Thus, it is very important to identify exact BMSC subpopulations that dismiss or increase during aging. Recent studies have been devoted to identifying some elderly BMSC subpopulations. Liu et al. have reported that a Sca1^+^ BMSC subpopulation from aged mice exhibited lower paracrine support for retinas than a Sca1^+^ BMSC subpopulation from young mice [60], and injecting young Sca-1^+^ BMSCs into 18-month-old mice through the tail vein can increase brain-derived neurotrophic factor (BDNF), ciliary neurotrophic factor (CNTF), FGF2, and insulin-like growth factor 1 (IGF-1) expression, downregulation of the apoptotic protein Bax with upregulation of the antiapoptotic protein Bcl2 to attenuate aging-related retinal degeneration ultimately [60]. Other studies have indicated impaired osteogenic potential of Prx1^+^ SSCs in aging mice because the loss of the peroxlsome proliferator-activated receptor-g coactivator-1 a (PGC-1α), an expression in aging Prx1^+^ SSCs, and overexpression of PGC-1α in SSCs reversed the unbalance of SSC osteo-adipogenic differentiation during aging [61]. Wang et al. have confirmed that alpha-ketoglutarate (αKG) can rejuvenate the osteogenic capacity of LepR^+^ BMSCs and ameliorate age-related osteoporosis by decreasing the accumulations of H3K9me3 and H3K27me3, and subsequently upregulates BMP signaling and Nanog expression [62]. Recently, a study by Yue revealed that LepR^+^ BMSCs were decreased with aging and enhanced adipogenesis in bone marrow, and downregulated HSC-supportive Kitl and Cxcl12 expression derived from LepR^+^ Notch3^+^ BMSCs [63]. Zhong et al. have found that the great expansion of LepR^+^ marrow adipogenic lineage precursors (MALPs) in 16-month-old mice might explain why there is more adipose tissue accumulation with aging [19].

In addition to aged mice, the study of Zhu et al. recently investigated the heterogeneity of BMSCs (CD29^+^ CD44^+^ CD90^+^ CD105^+^ CD34^−^ CD45^−^ HLA-DR^−^) from an 85-year-old human by sc-RNAseq [64]. They divided the BMSCs populations into three clusters according to the expression of functional genes [64]. Cluster 1 enriched the expression of cell self-renewal including cell division cycle-associated 5 (*CDCA5*) [65] and V-Myb avian myeloblastosis viral oncogene homolog-like 2 (*MYBL2*) [66], the cell metaphase–anaphase transition-related regulator family with sequence similarity 64 (*FAM64A*) [67], the integrity of the human centromere DNA repeats related to centromere protein A (*CENP-A*) [68], cell proliferation-related progestin and adipoQ receptor family member 4 (*PAQR4*) [69], DNA replication and repair related histone chaperone anti-silencing function 1B (*Asf1b*) [70], and chromatin assembly factor-1 (*CAF-1*) [71] and high mobility group protein 2 antibody (*HMGB2*) [72]. Cluster 2 is high expression multidirectional differentiation of BMSCs, including chondrogenesis-related transglutaminase 2 (*TGM2*) [73] and *COL11A1* [74], osteogenesis-related nuclear factor of activated T-cells (*NEAT1*) [75] and Type V collagen [76]. Cluster 3 enriched the expression of secretory factors to participate in immune regulation and damage repair including microbial killing and innate immunity-related *Cyba* [77], regulation of immunity and inflammation-related tissue inhibitor of metalloproteinase-1 (*TIMP-1*) [78] and annexin A1 (*ANXA1*) [79], cell migration and wound repair-related lumican (LUM) [80], and dermatopontin (*DPT*) [81], regulating cytokine secretion-related endoplasmic reticulum resident protein 44 (*ERp44*) [82], and protein import into endoplasmic reticulum-related heat shock protein family A, member 5 (*HSPA5*) [83].

Taken together, specific BMSC subpopulations exhibit changed numbers and impaired osteogenesis, and enhanced adipogenesis during aging (Table 1). For example, Sca1^+^, Prx1^+^, LepR^+^, LepR^+^ Notch3^+^ BMSCs of aging mice were all declined and exhibited impaired paracrine support for retinas and HSC niche, while LepR^+^ MALPs were increased in aging mice and contributed to adipogenesis. Moreover, elderly human BMSC populations can be divided three populations, including self-renewal, multidirectional differentiation, and immune regulation and damage repair.

### 2.2. Environmental Stresses

Apart from the different development stage, the heterogeneous BMSCs are also important for bone to react to different environmental stresses. Here, we review the functional heterogeneity of BMSC subpopulations under various environmental stresses, including loading, microgravity, hypoxia, irradiation, and PTH treatment (Table 1).

#### 2.2.1. Loading

Mechanical loading stimulates bone formation while BMSC subpopulations can contribute to this process. Many recent studies have focused on which specific BMSC subpopulations sense the signal of mechanical loading to osteogenesis. Cabahug-Zuckerman et al. have reported that mice Sca-1^+^ Prx1^+^ subpopulations participate in load-induced periosteal bone formation [84,85]. Mice without periosteal Prx1^+^ MSC exhibited mechanically impaired response capacity and declined bone formation and mineralization, accompanied by lower levels of osteogenic markers expression [86]. The research of Zannit has proposed that the Osx^+^ BMSC subpopulations also can be activated and contribute to loading-induced periosteal bone formation in both male and female mice [87]. Leucht et al. have reported that stromal cell-derived factor-1 (SDF-1, also known as CXCL12) was expressed in marrow cells, participating in load-induced bone formation [88], and subsequent studies found peri-sinusoids CAR cells [11,38]. The most recent work of Shen has reported that peri-arteriolar LepR^+^ osteolectin^+^ subpopulations can sense mechanical stimulation by mechanosensitive ion channel PIEZO1 to osteogenesis [89]. They observed that the number and osteogenic potential of LepR^+^ osteolectin^+^ BMSC subpopulations were increased when the mice were under voluntary running, while it would be the opposite when the mice were under hindlimb unloading. It has to be stressed that LepR^+^ osteolectin^+^ BMSC subpopulations just give rise to bone, not fat [89].

In sum, many specific BMSC subpopulations of mice are sensitive to loading-induced bone formation. In detail, periosteal Sca-1^+^ Prx1^+^, Osx^+^ BMSCs, peri-sinusoids CXCL12^+^ BMSCs, and peri-arteriolar LepR^+^ osteolectin^+^ BMSCs can sense mechanical loading to participate in bone formation.

#### 2.2.2. Microgravity

Studies of astronauts in spaceflight and animals exposed to simulated microgravity revealed that exposure to microgravity induces trabecular bone loss and increased adipogenesis in the bone marrow [104,105]. As opposite as mechanical loading stimulates bone formation, it remains to be shown if there are also specific BMSC subpopulations to sense the microgravity to induce bone loss and promote adipogenesis. The earlier finding showed that mice subjected to hindlimb unloading showed a declining number of the Sca^+^ CD90.2^+^ BMSC subpopulations [90]. Other research has identified Lin^−^ LepR^+^ SSCs under mechanical unloading are more quiescent and exhibit lower bone anabolic and neurogenic pathways [91]. Shen et al. have reported that the number and osteogenic potential of peri-arteriolar LepR^+^ osteolectin^+^ BMSCs were impaired when the mice were under hindlimb unloading [89].

All in all, Sca^+^ CD90.2^+^, Lin^−^ LepR^+^, and LepR^+^ osteolectin^+^ BMSC subpopulations each showed decreased number and osteogenic potential under hindlimb unloading in mice.

#### 2.2.3. Hypoxia

Bone marrow is a naturally hypoxic environment. Imaging analysis of BMSCs by cell surface markers found that these cells are located in regions where oxygen tension ranges from 4 to 1% [11,53]. How do the heterogenous BMSC subpopulations adapt physiologic oxygen tensions in the bone marrow to proliferation or differentiation? The previous evidence indicated that the number of CD13^+^ CD29^+^ CD44^+^ CD73^+^ CD90^+^ CD105^+^ CD151^+^ CD34^−^ BMSCs colonies developed was higher under hypoxia (3% O2) [92]. The recent study of Guo showed that PDGFRα^+^ and LepR^+^ BMSC subpopulations exhibited increased proliferation under 1% O2, while a 10-fold reduction in CD45^+^ hematopoietic cells [93]. In addition to proliferation, hypoxia also can influence specific BMSC subpopulations differentiation. For example, low oxygen tension stabilizes hypoxia-inducible factor alpha (HIFα) in SP7^+^ BMSCs (also known as Osx^+^ BMSCs) to stimulate osteogenic differentiation through direct activation of key glycolysis enzymes like pyruvate dehydrogenase kinase 1 (PDK1) [94]. Moreover, Nick et al. have reported that primary mouse skeletal stem cells (7AAD^−^ CD45^−^ Ter119^−^ Tie2^−^ CD51^+^ CD105^−^ CD90.2^−^ CD249^−^ CD200^+^) under 2% O2 were prone to chondrogenic differentiation via upregulating the expression of Sox9 while inhibiting fatty acids β-oxidation level [95].

Overall, BMSC subpopulations exhibit increased proliferation and enhanced osteo-chondrogenesis under hypoxia. CD13^+^ CD29^+^ CD44^+^ CD73^+^ CD90^+^ CD105^+^ CD151^+^ CD34^−^, PDGFRα^+^, and LepR^+^ BMSC subpopulations exhibited increased proliferation, while SP7^+^ and 7AAD^−^ CD45^−^ Ter119^−^ Tie2^−^ CD51^+^ CD105^−^ CD90.2^−^ CD249^−^ CD200^+^ BMSC subpopulations were prone to osteogenic and chondrogenic differentiation via upregulating glycolysis or fatty acids β-oxidation, respectively.

#### 2.2.4. Irradiation

Irradiation is a common method to eliminate the resident bone marrow hematopoietic stem before transplanted cells reconstitute hematopoiesis [96]. Recent efforts have found that irradiation can also alter BMSC subpopulations. For example, irradiation can lead to some BMSC subpopulations loss including LepR^+^ and Nestin^+^ subpopulations [96]. In contrast, a subpopulation expressing Ecto-5′-nucleotidase (CD73) was retained in mice after irradiation conditioning and a specific CD73^+^ NGFR*^high^* BMSC subpopulation contributes to HSPC engraftment and acute hematopoietic recovery via express various hematopoiesis supporting related factors like SCF, SDF1, Kit-L, Osteopontin, interleukin (IL)-3, IL-6, IL-7, and tumor necrosis factor alpha (TNF-α) et al. [96]. Zhong et al. has found that peri-arteriolar LepR^+^ MALPs express high myofibroblast genes (such as *Myl9*, *Col9a1*, *Col10a1*, et al.) to participate in bone marrow repair after radiation damage [19]. Recently, Yue’s group has revealed that osteogenesis subpopulations of LepR^+^ BMSCs were dramatically expanded in irradiation-conditioning mice, and the subpopulation-specific express transcriptional factor Npdc1 and Hoxb2 [63]. Importantly, the research of Xiang has studied the response of human bone marrow stromal cells to irradiation in vitro [97]. They found that human BMSCs labeled by CD73^+^ CD90^+^ CD105^+^ CD14^−^ CD34^−^ CD45^−^ HLA-DR^−^ showed senescent and impaired immunomodulation capacity after irradiation [97].

Taken together, irradiation perturbates the number, osteogenic capacity, and immunomodulation capacity of specific BMSC subpopulations. LepR^+^, Nestin^+^ BMSCs were lost after irradiation, CD73^+^ NGFR*^high^*, LepR^+^ MALPs, LepR^+^ BMSCs with high expression of Npdc1/Hoxb2 were expanded and contribute hematopoietic and bone marrow repair. Additionally, CD73^+^ CD90^+^ CD105^+^ CD14^−^ CD34^−^ CD45^−^ HLA-DR^−^ human BMSCs exhibited senescent impaired immunomodulation capacity after irradiation.

#### 2.2.5. PTH

PTH is a potent bone anabolic hormone [106]. The heterogeneous BMSC subpopulations have been recognized in PTH-induced bone formation. There was a study that identified quiescent LepR^+^ BMSCs located in healing sockets tissue of teeth [98]. The subpopulations can be activated by tooth extraction and contribute to extraction socket healing and alveolar bone regeneration of extraction sockets via response to PTH/PTH1R signaling [98]. Similarly, Yang et al. have used mouse genetic lineage tracing, which indicated that iPTH treatment increased the number of LepR^+^ BMSCs and the capacity of differentiating into type I collagen (Col1)^+^ mature osteoblasts [99], accompanied by increasing the expression levels of osteogenic markers *SP7/Osx* and *Col1* while decreasing the expression of adipogenic markers *Cebpb*, *Pparg*, and *Zfp467* [99]. They further found iPTH treatment can also inhibit 5-fluorouracil- or ovariectomy (OVX)-induced adipogenesis of LepR^+^ BMSCs and promote osteogenesis in bone marrow, even under adipocyte-induced conditions [99]. A further mechanism of PTH-induced LepR^+^ BMSCs osteogenesis may be due to promoting osteolectin expression [100]. Another different insight proposed that iPTH induced bone formation of LepR^+^ BMSCs by increasing numbers of type H endothelial cells (which are labeled by Edm*^high^*/CD31*^high^*) and mobilizing LepR^+^ cells from these vessels close to the bone surface [101]. In addition, other studies have found that LepR^+^ Runx2-GFP*^low^* exhibited enhanced Runx2 expression and more multilayered structures formation near the bone surface after PTH administration, and multilayered cells express Osterix and Type I collagen α, ultimately leading to the generation of mature osteoblasts [102].

In sum, PTH treatment can increase the number of LepR^+^ and LepR^+^ Runx2-GFP*^low^* BMSC subpopulations and skewed their lineage differentiation toward osteoblasts may by promoting osteolectin expression or increasing numbers of type H endothelial cells to make sure LepR^+^ cells transfer to the bone surface.

## 3. Functional Heterogeneity of BMSC Subpopulations in Pathology

Heterogeneous BMSC subpopulations contribute to the regulation of physiological processes but also to fracture, osteoporosis, and various disease processes. The declining number of BMSCs populations and impairing capacity of regeneration and osteogenesis are closely related to various bone-related pathological conditions. Targeting specific BMSC subpopulations offers potentially innovative therapeutic strategies for bone-related diseases. In this part, we summarize the functional heterogeneity of BMSC subpopulations under pathological conditions including fracture, osteoporosis, heterotopic ossification, obesity, and acute myeloid leukemia (AML) (Table 2).

### 3.1. Fracture

Fracture activates multiple BMSC subpopulations with high clonogenic capacity as well as tri-lineage differentiation potential that contributes to bone remodeling in fracture healing [107]. Mx1^+^ aSMA^+^ periosteal SSCs were shown to promote fracture healing in a CCR5-dependent manner [4]. Grem1^+^ and LepR^+^ periosteal cells subpopulations are expanded in response to bone fracture [16,27]. Colnot and colleagues confirmed that periostin^+^ periosteum stem cells (PSCs) possess high bone regenerative potential, and loss of periostin in PSCs exhibited a decline in number and osteogenic capacity [107]. Moreover, Debnath et al. used *CTSK-Cre*; *mTmG* reporter mice to trace BMSCs and PSCs which participate in the recovery process, and they found that Cathepsin K (CTSK)^+^ PSCs promote fracture healing, but no effect on hematopoiesis support [108]. Osx^+^ BMSCs support rapid periosteal angiogenesis at the time of bone injury and woven bone formation during fracture repair by expressing VEGFA [109]. It has to be stressed that VEGFA from another cell source like Dmp1^+^ mature osteoblasts/osteocytes does not have the same effect [109]. Another study proposed that Osx^+^ BMSCs contribute fracture healing through invading blood vessels moving into fractured sites [34]. Recently, Yue’s group has revealed that osteogenic subpopulations of LepR^+^ BMSCs were dramatically expanded in fracture mice, and the subpopulations specifically express transcriptional factors Npdc1 and Hoxb2 [63]. Currently, another research supports that Osx^+^ Sostdc1^(−/−)^ mice exhibited accelerated bone remodeling of the callus, suggesting that Sostdc1 may contribute to PSCs quiescence [110]. In addition to BMSCs contributing fracture healing, Zhong et al. have confirmed that Col10a1-Cre labeled hypertrophic chondrocytes can transdifferentiation to become osteoblasts participating in endochondral bone formation and fracture healing [111]. 

In summary, many kinds of specific BMSC subpopulations are activated and enhanced osteogenic capacity by fracture. Among these subpopulations, Mx1^+^ aSMA^+^, Grem1^+^, LepR^+^, Periostin^+^, and CTSK^+^ periosteal stem cells, as well as Osx^+^, LepR^+^, Osx^+^ Sostdc1^(−/−)^ BMSCs contribute fracture healing via increasing the BMSC number and osteogenesis. Additionally, Col10a1-Cre-labeled hypertrophic chondrocytes can also promote fracture healing via transdifferentiation to osteoblasts.

### 3.2. Osteoporosis

The impaired osteogenic capability of BMSCs is a major pathogenesis of osteoporosis [130]. Osteoporosis leads to increased bone fragility and occurrence of fractures, with limited effective prevention and treatment options [131]. Because of the heterogeneity of BMSC subpopulations, it is critical to understand the change in BMSC subpopulations during osteoporosis, and targeting a single specific BMSC subpopulation for intervention or transplantation may be more effective. Recent evidence supported that the ovariectomized (OVX) induced mice exhibited a declining number and osteogenic potential of Prx1^+^ SSCs, partly due to decreasing mitochondrial biogenesis and PGC1α level [61]. Other studies have found that upregulating microRNA-188 [112] or downregulating Kindlin-2 [113], NAD^+^ [114], and EGFR signal [115] in Osx^+^ BMSCs can lead to a reduction of osteogenesis in age-related osteoporosis or osteopenia. Furthermore, Jun treatment can relieve osteoporosis in mice through activating the osteogenesis capacity of Thy^+^/6c3^−^ BMSCs [116]. Pulsed electromagnetic fields (PEMFs) attenuated the senescence of LepR^+^ BMSCs to prevent bone loss in glucocorticoid-induced osteoporosis [117]. Mechanically, PEMFs trigger a high level of EZH2- H3K27me3 in LepR^+^ BMSCs for an anti-senescence effect [117].

Overall, Prx1^+^, Osx^+^ BMSCs exhibited lower osteogenic capacity in mice with osteoporosis, and active Thy^+^/6c3^−^ and LepR^+^ BMSCs by Jun addition or PEMFs can attenuate osteoporosis in mice.

### 3.3. Heterotopic Ossification

Heterotopic ossification (HO) is a form of pathological differentiation of BMSCs that occurs post a traumatic injury, usually accompanied by limiting motion in extremities and tissue pain [118]. Recent efforts led to identification of site-specific ectopic BMSC subpopulations. Previous data supported that Tie2^+^ endothelial precursors [132] or Tie2/vWF/VeCadherin-positive endothelium [133,134], Scleraxis-expressing(Scx^+^) tendon-derived progenitor [135], muscle-resident interstitial Prx1/Mx-1/PDGFRα positive populations [119,136,137], and peripheral progenitors labeled by odd-skipped related (*Osr1*, *Osr2*) genes [138] and engrailed1 (*En1*) [139] all can respond to a traumatic inflammatory trigger and contribute to heterotopic bone formation. Later evidence confirmed that Tie2^+^ progenitors in traumatic sites also co-express Osterix, SOX9, PDGFRα, Sca1, and S100A4 et al. MSC markers suggesting more MSC subpopulations participate in HO process [137,138,139]. The following study by Agarwal has reported that Prx1^+^ BMSCs were involved in HO development [119]. They further revealed that hypoxia-inducible factor-1α (Hif1α) is highly expressed in three separate HO mouse models (trauma-induced, genetic, and a hybrid model of genetic and trauma-induced HO) and promotes endochondral ossification of Prx1^+^ BMSCs to form extraskeletal bone [119]. Meanwhile, pharmacologic inhibition of Hif1α using PX-478 or specific knockout Hif1α in Prx1^+^ BMSCs resulted in substantially decreased HO [119]. Another piece of evidence identified PDGFRα^+^ MSCs secret high-level VEGFA to induce heterotopic ossification [120]. Most recently, Pagani et al. used inducible lineage-tracing mouse (*Hoxa*11- CreER^T2^; *ROSA26*- *LSL*- *TdTomato*) to establish a mouse HO model, and they found that Hoxa11 can successfully label HO progenitors in the zeugopod and Hoxa11^+^ BMSCs can be activated to undergo differentiation toward chondrocytes and osteoblasts [118].

In sum, earlier studies confirmed that Tie2^+^ endothelial precursors, Tie2/vWF/VeCadherin-endothelium, Scx^+^ tendon-derived progenitor, muscle-resident interstitial Prx1/Mx-1/PDGFRα positive populations, and Osr1/Osr2/En1 positive peripheral progenitors were all involved in the HO process. Recent evidence suggested Prx1^+^ BMSCs and PDGFRα^+^ MSCs contribute to HO via upregulating Hif1α or increasing VEGFA secretion, respectively. Additionally, Hoxa11 also can specifically label HO progenitors in the zeugopod.

### 3.4. Obesity

Obesity result from disturbed osteo-adipogenic differentiation of BMSC subpopulations and presents emerging challenges for our society. Interfering with dysregulated BMSC subpopulations in the obese will open new perspectives to prevent obesity. The research of Tencerova investigated BMSCs of 54 men divided into lean, overweight, and obese groups according to their basis of BMI, and found that obese BMSCs exhibited skewed lineage differentiation toward adipogenesis and enhanced the genes expression of glycolytic and oxidoreductase activity [121]. Importantly, in contrast to peripheral adipose tissue-derived stromal cells (AT-MSCs), obese BMSCs exhibited higher insulin signaling, accelerated senescence phenotype, and expansion of insulin receptor positive (IR^+^) and LepR^+^ cells in bone marrow [121]. This evidence may partly explain why bone loss occurs in the obese. Picke et al. also observed that the number and differentiation capacity of CD45^−^ Sca-1^+^ BMSCs declined under obese conditions, whereas the total number of MSCs was not changed [122]. They further confirmed dysregulation of CD45^−^ Sca-1^+^ BMSCs may be due to reducing Thy-1 expression (CD90) which keeps stable differentiation of MSCs [122]. Consistently, Thy-1-deficient mice exhibited smaller bone volume and lower bone formation rate with increased cortical porosity, ultimately resulting in lower bone strength, while body weight, subcutaneous/epigonadal fat mass, and bone fat volume were all increased [122]. Additionally, Ambrosi et al. observed that dipeptidyl peptidase-4 (DPP4) increased in CD45^−^ CD31^−^ Sca1^+^ CD24^+^ BMSCs in obesity mice, and blunting DPP4 would recovery osteogenesis of CD45^−^ CD31^−^ Sca1^+^ CD24^+^ BMSCs and improve obesity [123].

Taken together, obese BMSC subpopulations exhibit lower osteogenic potential and expansion of insulin receptor-positive (IR^+^) and LepR^+^ cells in bone marrow in humans. CD45^−^ Sca-1^+^, Sca1^+^ CD24^+^ subpopulations may exhibit impaired osteogenesis capacity in obese mice through downregulating Thy-1 expression or upregulating DPP4 level.

### 3.5. Acute Myeloid Leukemia (AML)

As mentioned above, multiple heterogeneous BMSC subpopulations serve as regulators of hematopoiesis [140] and the dysfunction of some contribute myelodysplasia and leukemia [141,142,143]. AML is hematopoietic malignancies associated with mutations in hematopoietic stem and/or progenitor cells [144]. During AML development, the BMSCs change substantially, like decreasing total BMSCs numbers, polygonal or irregular cell shapes, and abnormal multi-differentiation potential [145,146]. Other studies have reported that peri-arteriolar Nes^+^ BMSCs reside both in AML patients and in MLL-AF9 mice [124,125] and this subpopulation exhibited abnormal proliferation and differentiation coinciding with depletion of the quiescent Nes*^peri^* population, and establishes an abnormal niche which trigger HSC exhaustion [124] maybe through reducing expression of VLA-4, VCAM-1, CXCL12, Ang-1, SCF, and TGFβ-1 [126] and increased expression of OPN. Concurrently, Nes^+^ BMSCs also exhibited high metabolic status to enhance leukemic stem cells (LSCs) antioxidant defense against oxidative stress and survive under chemotherapy [125]. The recent study of van Gastel reported that CD45^−^ Ter119^−^ CD31^−^ LepR^+^ BMSC subpopulations can convert glutamine into aspartate via expressing a high level of aspartate-glutamate transporter SLC1A3, fuel AML cells for pyrimidine generation and protects them against chemotherapy-induced cell death [127]. Moreover, Yuan et al. observed Osx^+^ BMSCs were expanded in mice implanted with AML cells, but endosteum and trabecular bone were reduced, thus suggesting osteoprogenitor cells under AML microenvironment cannot fully differentiate mature osteoblasts [128]. Similarly, DAPI^−^ CD45^−^ CD235a^−^ CD31^−^ CD146*^low/+^* CD271^+^ BMSCs in AML patients also exhibited lost quiescence, significant expansion, and impaired HSC-niche-supporting capacities, lower osteogenesis by downregulating cytoplasmic β-catenin [129].

In sum, AML decreases BMSC subpopulations number, alters cell shape, and impairs multi-potential. In detail, Nes^+^ subpopulations in AML mice and humans exhibit abnormal proliferation and trigger LSCs exhaustion; while CD45^−^ Ter119^−^ CD31^−^ LepR^+^ subpopulations protect AML cells, avoiding chemotherapy-induced death by expressing high level of SLC1A3. Additionally, Osx^+^ subpopulations and DAPI^−^ CD45^−^ CD235a^−^ CD31^−^ CD146*^low/+^* CD271^+^ BMSC subpopulations’ expansion in AML mice and patients both exhibit lower osteogenesis.

## 4. Unresolved Questions, Challenges, and Potential Opportunities

BMSCs are heterogeneous cell populations involved in bone development, homeostasis, and various pathological conditions. Recent efforts have reported functional heterogeneity of BMSC subpopulations under various physiological and pathological states driven by application of flow cytometry, lineage tracing, and scRNA- seq. However, to date, our understanding of the complex heterogeneous composition of BMSCs still remains incomplete, especially lacking data for humans. In addition, it is critical to explore composition and function of heterogeneous BMSC subpopulations at different developmental stages, or investigate the mechanism of how single specific BMSC subpopulations respond to various environmental stress (like loading, microgravity, hypoxia, and other physical factors) or diseases (fracture, osteoporosis, obesity et al.). These issues still need a comprehensive study. In subsequent studies, a novel mice model which allows us to observe heterogeneous BMSCs’ variety in their native niche will be available and will be a key tool in this effort. Moreover, new technological application like computational trajectory inference enables us to reconstruct cell state dynamics through analyzing data from scRNA-seq. Importantly, the recent study of Lange et al. proposed a new approach, CellBank (https://cellrank.org, accessed on 21 August 2022), which combines the robustness of trajectory inference with directional information from RNA velocity, automatically detects initial, intermediate, and terminal populations, predicts fate potentials and visualizes continuous gene expression trends along individual lineages [147]. CellBank may be an effective tool to study heterogeneous BMSC subpopulations and revise previous dogma. Identifying the true single BMSC subpopulation under a specific situation offers a potentially innovative therapeutic strategy and improves BMSC effectiveness in clinical application.

In investigating the functional heterogeneity of BMSC subpopulations under different physiological and pathological conditions, researchers also face some challenges. First, dividing BMSCs into functionally distinct sub-groups based on cell surface markers is not appropriate, as their expression is overlapping across subpopulations. For example, LepR^+^ BMSCs overlap with Nestin-GFP*^low^* cells [40,41], Cxcl12-abundant reticular (CAR) cells [44], and Osx^+^ BMSCs [39], and these populations both possess multi-potential and support HSC. Thus, there needs to be a more standardized functional characterization of potential BMSC subpopulations. When researchers found a new BMSC subpopulation with a specific function, do not rush to give this subgroup a new name, just check whether the subpopulation co-expresses known stem cell surface marker through flow cytometry, or use the lineage-trace mouse model to compare the overlap and similarity between the subpopulation you found and previous progenitors. Second, identify a specific BMSC subpopulation which indeed plays a critical role under certain physiology and diseases, because too many BMSC subpopulations were identified; but which one is more potent to target or transplant to treatment diseases? Maybe we should make a cross-sectional comparison of the treatment effects of BMSC subpopulations in vivo; or, we can use the CellBank approach to detect initial, intermediate, and terminal populations, predict fate potentials, and visualize continuous gene expression trends along individual lineages. Third, the process of isolated specific BMSC subpopulations for in vitro study may lose some natural characteristic of BMSCs. Developing new animal models with intravital labeling BMSC subpopulations by specific stem cell markers may provide a new insight for direct visual analysis of distribution of different BMSC subpopulations in their native microenvironment and under stress. Another methodology that proves helpful to understanding of BMSC heterogeneity in vivo is the development of a device for the isolation of BMSC subpopulations while preserving their natural marker and function, thereby permitting further analysis.

Finally, mounting evidence suggests the energy metabolism is critical for BMSC function [148], whether heterogeneous BMSC subpopulations exhibit distinct potential due to metabolic heterogeneity. A recent paper from Joffin et al. [149] highlights the importance of mitochondrial metabolism in two PDGFRβ^+^ adipocyte subpopulations’ fate, including adipogenic progenitor cells (APC) and fibro-inflammatory precursors (FIP), which are two with a distinctive metabolism in white adipose tissue [149]. In the following years, a comprehensive understanding of whether metabolic heterogeneity among BMSC subpopulations is involved in health and diseases, and whether pharmacological targeting of the dysregulated metabolic pathways in specific BMSC subpopulations can restore their function and remission diseases, will be of considerable interests.

## Figures and Tables

**Table 2:** Functional heterogeneity of BMSC subpopulations in pathology.

Pathological Conditions	BMSC Subpopulations	Functions	References
Fracture	Mx1^+^ aSMA^+^, Grem1^+^, LepR^+^, Periostin^+^, and CTSK^+^, Osx^+^, Osx^+^ Sostdc1^(−/−)^	Expands and actives osteogenesis in response to bone fracture	[4,16,27,107,108,109,110,111]
Osteoporosis	Prx1^+^, Osx^+^, Thy^+^/6c3^−^ and LepR^+^	Exhibits lower osteogenic potential: Prx1^+^, Osx^+^	[61,112,113,114,115,116]
		Actives Thy^+^ /6c3^−^, LepR^+^ can treat osteoporosis	[116,117]
Heterotopic ossification	Prx1^+^, PDGFRα^+^, Hoxa11^+^	Upregulates Hif1α or increases VEGFA secretion to promote endochondral ossification to form extraskeletal bone	[118,119,120]
Obesity	IR^+^ and LepR^+^ in obese bone marrow; CD45^−^ Sca-1^+^, Sca1^+^ CD24^+^	Exhibits lower osteogenesis potential and dysregulated metabolism;	[121]
		Impairs osteogenesis capacity through dowregulating Thy-1 expression or upregulating DPP4 level	[122,123]
AML	Nes^+^, CD45^−^ Ter119^−^ CD31^−^ LepR^+^, Osx^+^, DAPI^−^ CD45^−^ CD235a^−^ CD31^−^ CD146*^low/+^* CD271^+^	Decreases BMSCs number, altered cell shape, and impairs multi-potential	[124,125,126,127,128,129]
